# Indents

**Published:** 1916-06

**Authors:** 


### INDENTS

**☞ Brief Articles of Technical or Scientific Value will receive notice and comment. Contributions invited.**

*Making Dies with Heavy Undercuts.* After impression is made apply separating medium as usual, then pour up the under-cut portions of the impression first and remove them from the impression and trim so they will present smooth surfaces. Replace in the impression and apply separating medium and pour the remainder of the impression; when the impression has been removed the model will be in several pieces according to position of undercuts. Assemble the parts of the model and pack the sand mould. In removing the model from the sand it can be taken out in sections without disturbing any part of the sand mould. The die metal then is poured and a perfect die is secured.—J. C. Linsley, in *Digest.*

*"Dental Hygienists."* More and more the importance of dental hygiene, particularly for school children, is being recognized. In a recent address at the Forsyth Dental Infirmary for Children, the director of that institution emphasized the dangers of poor teeth in school children both for themselves and for others. "All must share in the morbid pathological conditions of the neglected child, and this is particularly true concerning tooth and mouth conditions. The time is rapidly approaching when a child will not be allowed to enter a public school until he is physically fit." Even disease of the first teeth, which have been too often disregarded because of their transient nature, is capable of producing lasting effects upon the child. When the temporary teeth are lost prematurely, the permanent teeth erupt unassisted into various conditions of irregularity and malocclusion, which greatly interfere with the growth of bones of the head. Deformities of the nose and mouth may result, which interfere with respiration and with voice production. Absorption from septic processes in the mouth has been much discussed of late.

Granting the truth of these facts does not settle the problem. The question of how to get so many thousands of mouths cared for, presents a number of difficulties. The interest recently aroused in dental hygiene has flooded the dentists with work. Many people can not afford regular dentists' fees for cleaning, and the elementary part of the work, which is sufficient for the teeth of many children, should not require the time of a dentist.

Laws dealing with this situation have been enacted in Massachusetts and Connecticut, and now the Governor of New York State has recently signed a bill which provides for the training, registration and licensing of the "dental hygienist." The new law provides that any dental dispensary or infirmary, legally incorporated and maintaining a proper standard and equipment, may establish for women students a course of study in oral hygiene. One year's attendance at high school and one year's training in oral hygiene are required to become a licensed "dental hygienist." The person so licensed "may remove lime deposits, accretions, and stains from the exposed surface of the teeth, but shall not perform any other operation on the teeth or tissues of the mouth."

It is to be hoped that plenty of "dental hygienists" will be developed and that the portion of the public, who heretofore have been unable to afford dentists' services save for the worst toothaches, will appreciate the value of clean mouths and avail themselves of the opportunity.—*Boston Medical and Surgical Journal.*

*Dental Disaster.* Energetic internists are grasping at infected tonsils and teeth with an alarming enthusiasm. No doubt these organs have been guilty of many remote infections. The tonsils seem to be quite rightly accused and convicted as primary offenders. The tonsils are wholly within the realm of medicine, and physicians need not argue tonsillar guilt with another profession. But the teeth—poor things—are being accused by physicians and defended by dentists. The dental radiograph has stepped in to promote the physician's case and to confuse the dentist. There is no doubt but that pathologists have shown that organisms, capable of remote infections, are found about the apices of diseased teeth. Consequently the physician is prepared to sacrifice the offending teeth immediately with the same assurance that he sacrifices tonsils, appendices and gall-bladders. But the dentist is not ready to see his pets sacrificed without a more thorough trial. He argues that infection about the teeth can be controlled by conscientious and painstaking dental surgery and that carelessly to extract these important organs places the individual at a distinct digestive disadvantage.

There appears to be little doubt, and dental radiography seems to prove, that there are serious infections about teeth which have undergone improper technical dental treatment. Also it is true that dangerous granulomas arise at the apices of injured teeth without previous dental attention. Many contributions (notably Rhein's) appear to prove that these granulomas may be completely healed by proper aseptic root-fillings. Is it necessary then to sacrifice teeth and force the patient to bridges and plates? It spoils two good teeth to anchor a bridge, and unless the anchor teeth are aseptically prepared the patient has two potential sources of infection where one previously existed. Plates may be uncomfortable, distort taste impressions and require unceasing attention to cleanliness. Bridges and plates, therefore, interfere with digestion and consequently the health and happiness of the patient. The human race as it is constituted today can not hope to avoid the route of the Dinosaurus if the teeth are to be continually sacrified.

The teeth must be preserved and used for their original purpose of mastication or they will contrive to make trouble. Perhaps the increase in soft prepared foods for children prevents the development of sound teeth. It requires exercise to develop muscle and study to develop brains and, likewise, mastication to develop healthy teeth. Otherwise muscles become flabby, brains stagnate, and teeth lack resistance and become soft, inviting infection. Again, dentists have not generally appreciated surgical cleanliness—asepsis. They are where surgery was twenty-five years ago in the antiseptic stage. Dentists still seal antiseptic preparations in root canals to eradicate infections. This is contrary to accepted surgical authority.

It would seem that it is necessary to inject much which is known in surgical pathology into dental teaching and also teach dentists the principles of surgical asepsis. Failure along these lines has forced physicians to advise the sacrifice of teeth. The conflict is still waging.—Dr. Edward H. Skinner, *Interstate Medical Journal.*

*Matrix Using Celluloid.* Lock the strip together by cutting on opposite sides, then heat small serrated flat nose pliars and fuse the celluloid by pinching with the heated pliers. The celluloid in Kodak films is suitable for this work when the coating is removed. To remove coating wash in hot water.—E. T. Evans, in *The Dental Review.*

*Some Dental Uses for Rice Flour.* To make this preparation of rice flour, take impression plaster of Paris 4 parts; rice flour 1 part; each to be added by measure; mix thoroughly. Place the powder in a box and label it "Crown and Bridge Impression Plaster." When preparing to take impression add a little more salt to the water than you would to plaster of Paris impression, as the rice flour slightly retards the crystallization of the plaster. After taking the impression, stain and pour model for crown or bridge. When model is hard, place model and impression in boiling water, and the impression will gradually disintegrate into small flakes, leaving a perfect model, crowns and teeth intact. When the impression starts to disintegrate in the boiling water, a sharp pointed instrument may be used to remove the soft parts of the impression, thus hastening the separation of the impression from the model.

No injury to the model will take place in the hot water, if the model has had time to become thoroughly hard.

Rice flour is not affected by cold or luke-warm water. Hot water causes the rice granules to increase in size, disintegrating the plaster impression.

Rice flour is a good substitute for precipitated chalk for cleaning the teeth. It adheres to the engine brush much better than precipitated chalk and is not as abrasive. The addition of two or three drops of ammonium biflorid to the rice flour will be found an aid in cleaning tobacco stain from the teeth.—C. H. Woolgar, in *Summary.*

*Making Injections for Conductive Anesthesia*

After you get the needle deep into the tissues, do you ever forget which side of the needle the bevel is out? As this is important to know while passing the needle along the surface of the bone, it is of great assistance to have a marking on the exposed instrument that will, at all times, indicate the exact position of your bevel.

Take a round bur and make a pit on one of the flat surfaces of the octagon hub of your needle. When you put the needle into the hub always see that the bevel on your needle corresponds to the pit made on the hub. This will tell you where your bevel is at all times.—J. M. Prime, in *Nebraska Dental Journal.*

*Wax Model Additions.*

It is well to have in the case wax of two quite different colors. Sometimes you wish to build out an inlay model for additional contour, or better occlusion. If you make the addition with wax of a different color you can see exactly where and how much you add.—Garrett Newkirk, in *Review.*

*Devitalization of Deciduous Teeth.* Every dentist is daily confronted with the perplexing problems of badly decayed deciduous teeth, in which, in a great number of cases, devitalization is the only alternative. Every modern dentist believes in preserving these teeth until the eruption of their successors. But the question arises how to quickly and painlessly accomplish this devitalization without overtaxing the little patient.

A method which is very successful is as follows: When devitalization becomes necessary, use mucous anesthesia. First, paint gum around tooth with iodin to promote asepsis, and see that all instruments are perfectly sterile. Then, with hypodermic syringe, using any good local anesthetic, inject in interproximal spaces on either side of tooth, carrying needle as near to apex of root as possible, and anesthetize the area around apices of the roots. Now, the pulp tissue can quickly and painlessly be removed. After checking hemorrhage, place a bland antiseptic dressing in canals for four to six days. The roots may then be filled and the permanent filling inserted. This method is very practical, due to the soft, cancellous nature of the process and the fact that the apices of the roots are large, thus allowing perfect anesthetization of the pulp tissue.— Floyd E. Gibbin, in *Review.*

*The Use of Floss Silk in the Dental Toilet.* In the use of the floss silk, one should first rub tooth-powder into the dental interspaces and then pass the floss gently down to and under the gum margin of one tooth without forcing into it so as to produce mechanical injury. The floss should then be bent as nearly as possible half-way around that particular tooth, and then with a slight sawing motion be slowly wiped over the entire approximal surface and part of the buccal and lingual as the floss is drawn toward the occlusal and out between the teeth. Next the floss is passed into the same space, and the adjoining tooth cleansed. The performance of the act once a week should be supplemented by the use of a thin rubber band nightly, as it is soft and less injurious mechanically than the floss.—*Dental Record.*

*Protecting Gum Margin in Partial Dentures.* The clinical experience of all offers a convincing proof of the first and most important essential in design of a partial denture, or, for that matter, any kind of artificial restoration of lost teeth and associated tissues, namely: the *necessity for keeping all parts, be they clasps, bands, half-bands, hoods, saddles, bars, etc., away from the free margin of the gum, let alone sinking between the root and the free margin.*—W. E. Cummer, in *Oral Health.*

*The Use of Clasps.* Clasps should grasp the most bulbous part of the tooth and a little above and a little below it, because this gives the best hold, least strains the elasticity of the metal, and is usually the part with a thick covering of hard enamel that can perfectly well bear the friction.—Douglas Gabell, in *Dental Cosmos.*

*Carefulness in Diagnosis.* That dentist who is a member of a dental society, and is reckless and neglectful of instrument and digital cleanliness, is more of a menace to humanity than the clean dentist and advertiser, since the latter does not inflict a body injury even if he does exaggerate his ability and extract a high or unearned fee—while the filth-fingered society member, not only violates his code, but may by his shiftless methods induce a disease intensely worse than dental ailments. By mistake, I overlooked the fact that he did ask a question before he began to administer the gas. He asked her before he opened her mouth, ''Did you bring any money with you?'' She answered: ''Yes doctor.'' ''How much?'' he quickly asked, and she in nervous tones replied ''One dollar,'' holding the argent up before his eyes. After all was done, she handed the silver dollar to the lady assistant, who gave her twenty-five cents in change. By all that's holy, do you know of any person either in business or the professions who would risk taking you to the portals of death and be responsible for your safe return for the paltry sum of seventy-five cents? No wonder he could not take time; no surprise that he cared little about her name or address; the affair was but a passing incident in the life of the second-assistant, who though a graduate, with state license registered, received the remarkable salary of fourteen dollars per week. Less than bricklayers or carpenters receive. With these low professional fees (be they of the so-called ethical practitioner or the non-society man), these naturally go as the accompaniment of haste and neglect, and post operation difficulties are sure to follow. The money must be made as in bargain-counter trade by quick sales! This feature which is creeping into our professional service, causes the better class of people to doubt your ability, and in this doubt rests and resides the fear, that you would not be a careful man to entrust in a case of anesthesia—because you are too cheap. Take your time, but quickly, yet thoroughly investigate the case,—you need not learn the entire genealogical career of the family of the patient, yet in a pleasant confidence-begetting manner seat the patient, and look well after their capabilities of free breathing—note the heart action and if least in doubt, get stethoscopic record. Then with sterilized instruments, appliances and clean hands, proceed to administer your anesthetic. Nothing will drive away the fear of a patient quicker than to observe that the operator seems careful, cleanly and cheerful,—the three ''Cs'' which will dispel the dread of the dental operator. Take time, prepare your patient mentally and physically—it will pay you in the end and to be known as a cautious, clear-headed, capable and sympathetic operator, will bring you splendid financial returns. The Mayo Brothers, away up in the northwest, in the dinky city of Rochester, are attracting to their hospitals, patrons from every clime and government of the world. Why don't they come to the big city of Chicago, or why not go to New York City? I can not answer that, but I know that the world has learned of their carefulness, and a beaten track is being made to their forest home. They insist on preparation! They demand that the patient be thoroughly examined before an anesthetic is chosen; in fact, if it is possible, the patient is obliged to be at their hospital for at least a week, before the operation is attempted. By proper foods, natural and required liquids and scant medicines, they finally arrange to operate. Have you learned of the expert methods they employ in administering anesthetics? Have you heard of the remarkably few deaths from anesthetics, recorded in their tens of thousands of operations? The basis of all this success rests on the diligent concern they take in preparation of the patient.—Dr. B. J. Cigrand, in *Facts*.

*Dental Economies.* Dentists of the country waste more than $200,000 each year by not guarding the fragments of gold which fall during the mouldings, polishing and setting of crowns, bridges and inlays. This loss may be entirely eradicated by a little care and small initial expense.

Care of the office books will add to the bank account. I know of one practitioner who died leaving uncollected accounts on his books amounting to $80,000. Some were twenty years old. Dentists should refuse treatment to the "dead beat" but to the poor, never!—Dr. Hinman.

*The First Plaster Impression.* In the January, 1916 issue of *The Pacific Dental Gazette* I have read with interest a paper by Dr. L. P. Haskell, of Chicago, entitled "The First Plaster Impression and First Suction Plate." In this article Dr. Haskell states that he saw the first plaster impression made by his brotherin-law, in Boston, in 1844, and states that he would "be pleased to hear if anyone can antedate the above."

It is always interesting to see how the same idea will strike men widely separated at approximately the same time. Will you allow me to quote the following from a paper by Dr. Amos Westcott, of Syracuse, published in *The Dental Cosmos* for April, 1870, entitled "The Use of Plaster of Paris for Taking Impressions of the Mouth—Its History and Importance?" The part of this paper of especial interest is as follows:

"Up to 1844 the use of plaster by the dentist was confined to the making of his models, by pouring it into the impressions of the mouth, taken with wax, or in combination with sand, for holding together his work while soldering.

"*Taking impressions of the mouth with plaster* was (so far as I am aware) first attempted in March, 1844—or about *twenty-six years ago.* At this time Dr. E. J. Dunning and myself were partners in business, at Syracuse, and as full of enthusiasm as if we believed the philosopher's stone within our easy reach. Dr. D. had previously done some dental business in Montrose, Pa., and returned there at the time above indicated to fulfill some other engagements. While there he undertook a case of plate work, which I leave him, in his own language, to describe. In a letter from him, dated January 10, 1870, in reply to a request from me to give his recollections of the origin of this practice, he says: 'It was in the winter of 1843-4. I was at Montrose, Pa., attending to some professional engagements. I had previously undertaken to make a set of teeth for an old gentleman, with very poor success. The upper jaw—all of the teeth having been removed years before—had been reduced by absorption to its minimum size. It was very small and very flat, and what was worse had two flabby and easily removed masses of tissue in the palatine arch. I had made several trials with wax impressions, but could never succeed in obtaining a correct cast on account of the moving of these bags, under the slightest pressure. In those days, you know, we hated to give up a case and acknowledge ourselves unable to conquer it. Such a result seemed inevitable. I thought of the case all day, and finally arrived at the stage of dreaming of it. Early one morning the idea of using *plaster* suggested itself to me, and after a little thinking over the "modus operandi," I became satisfied that a valuable resource in this case, and perhaps in others, had been devised. The relief from my special dilemma was so great that I danced around my room for joy, and when ——— (a student) came in, I think my demeanor must have been more amusing than dignified. I think I *remember brandishing the poker around his head!* Well, this is all I know of the origin of taking plaster impressions. I obtained a better cast of old Mr. ——'s mouth than I had before had, but I doubt if, after all, the teeth answered. I do not doubt but you have made the process of taking plaster impressions so different from my first clumsy experiment, that I should fail to recognize it, except in idea. Very soon afterward I left for New York and have since devoted my time *exclusively* to the chair.'

"The above letter from Dr. Dunning is very satisfactory, as it fully confirms my own recollections upon the subject."

In view of the great importance of the plaster impression in dental prosthetics, I feel confident this bit of history in the above letter, written by my grandfather, will be found worthy of record in your journal.—Wm. B. Dunning in *Pacific Dental Gazette.*

